# Co-producing an intervention for tobacco cessation and improvement of oral health among diabetic patients in Bangladesh

**DOI:** 10.1186/s12903-021-01861-0

**Published:** 2021-10-12

**Authors:** Masuma Pervin Mishu, Helen Elsey, Arup Ratan Choudhury, Shahana Dastagir, Saeed Khan, Tania Tahsin, Hena Moni Suma, Rajesh Karmaker, Omara Dogar

**Affiliations:** 1 Department of Health Sciences, University of York, Heslington, York, YO10 5DD UK; 2 Department of Dentistry, Bangladesh Institute of Research and Rehabilitation in Diabetes, Endocrine and Metabolic Disorders (BIRDEM), Dhaka, Bangladesh

**Keywords:** Diabetes, Dental care, Barriers, Facilitators, Tobacco cessation, Intervention, Co-production

## Abstract

**Background:**

Tobacco consumption is a major risk factor for many diseases including diabetes and has deleterious effects on oral health. Diabetic patients are vulnerable to developing certain oral conditions. So far, no studies have attempted to co-develop a tobacco cessation intervention to be delivered in dental clinics for people with diabetes in Bangladesh.

**Aim:**

To co-produce a tobacco cessation intervention for people with diabetes for use in dental clinics in Bangladesh.

**Objectives:**

To assess: (1) tobacco use (patterns) and perceptions about receiving tobacco cessation support from dentists among people with diabetes attending the dental department of Bangladesh Institute of Research and Rehabilitation in Diabetes, Endocrine and Metabolic Disorders (BIRDEM) who smoke or use smokeless tobacco (ST) (2) current tobacco cessation support provision by the dentists of the dental department of BIRDEM (3) barriers and facilitators of delivering a tobacco cessation intervention at a dental clinic, and (4) to co-produce a tobacco cessation intervention with people with diabetes, and dentists to be used in the proposed context.

**Methods:**

The study was undertaken in two stages in the dental department of BIRDEM, which is the largest diabetic hospital in Bangladesh. Stage 1 (July–August 2019) consisted of a cross-sectional survey among people with diabetes who use tobacco to address objective 1, and a survey and workshop with dentists working in BIRDEM, and consultations with patients to address objectives 2 and 3. Stage 2 (January 2020) consisted of consultations with patients attending BIRDEM, and a workshop with dentists to co-produce the intervention.

**Result:**

All survey participants (n = 35) were interested in receiving tobacco cessation support from their dentist. We identified important barriers and facilitators to deliver tobacco cessation intervention within dental services. Barriers reported by dentists included lack of a structured support system and lack of training. As a facilitator, we identified that dentists were willing to provide support and it would be feasible to deliver tobacco cessation intervention if properly designed and embedded in the routine functioning of the dental department of BIRDEM. Through the workshops and consultations at stage 2, a tobacco cessation intervention was co-developed. The intervention included elements of brief cessation advice (using a flipbook and a short video on the harmful effects of tobacco) and pharmacotherapy.

**Conclusion:**

Incorporation of tobacco cessation within dental care for people with diabetes was considered feasible and would provide a valuable opportunity to support this vulnerable group in quitting tobacco.

## Introduction

Tobacco (both smoking and smokeless) use is a known risk factor for many chronic diseases, posing major health challenges and economic burden [1]. Smoking has been recognized as one of the biggest risk factors for oral diseases such as periodontitis (disease of tooth-supporting bone and soft tissue) and tooth loss [2, 3] Smoking accelerates periodontitis and reduces the response to dental treatment [4], as it impairs the periodontal tissue's ability to heal [5]. Smoking also causes discolouration of teeth and bad breath [6]. Smokeless tobacco (ST), particularly chewing tobacco, is also harmful for oral health. ST (particularly that consumed in South-East Asia (SEA)) contains harmful chemicals like tobacco-specific N-nitrosamines (TSNAs) [7], which are known risk factors for oral cancers and also increase the risk of different oral lesions, including potentially malignant disorders [8, 9].

Globally, 463 million people have diabetes and the majority of them are from the SEA region [10]. Diabetes is a major health challenge in SEA [11], with a regional prevalence of 8.8% of adults [12]. People with diabetes are more prone to certain oral diseases [13]. A review showed a higher prevalence of oral mucosal disorders in people with diabetes (45–88%) compared with the non-diabetic population (38–45%) [14]. Periodontitis is considered as the sixth most common complication of diabetes [15]. Evidence suggests that there is bidirectional association between periodontal disease and diabetes mellitus [16–19]. Treating periodontal disease can be influential in contributing to glycaemic control and vice versa [17]. As tobacco use increases the risk of both diabetes and oral diseases, tobacco cessation is particularly important in improving the general and oral health of people with diabetes. A review of more than 200 studies concluded that there is a clear need to increase the frequency of smoking cessation advice and counselling for people with diabetes [20]. The National Diabetes Education Program under CDC has identified specific providers, including dentists, to work together through interdisciplinary collaboration and to implement evidence-based strategies to ask, advise, and assist patients in reducing their risk behaviors and encouraging healthy behaviors, such as smoking cessation [21]. It is therefore important to offer tobacco cessation support to people with diabetes who use tobacco by using a tailored tobacco cessation intervention as part of their routine dental care.

People who are diagnosed with oral lesions or advanced periodontitis worry about developing oral cancer or losing their teeth and this can then lead them to alter their health behaviours positively [22]. This is known as a ‘teachable moment’. Dental professionals can use this opportunity to offer tobacco cessation advice [23], although this opportunity is rarely taken [24]. A systematic review by Holliday et al. (2021) provided evidence with moderate-certainty that tobacco abstinence rates increase in cigarette smokers if dental professionals offer behavioural support in conjunction with the provision of nicotine replacement therapy (NRT) or e-cigarettes, when compared with no intervention, usual care, or brief, or very brief advice only (RR 2.76, 95% CI 1.58–4.82) [25]. Interventions in the dental setting usually included a multi-component tobacco cessation interventions in conjunction with an oral examination [26]. Interventions included behavioural support in the form of brief advice, in combination with different intervention components such as video-based tobacco cessation with phone follow up [27–29], or referral to a quitline [30]. Some studies included the 5 A’s intervention (ask, advise, assess, assist, and arrange counselling with quitline referral as an option at the provider's discretion). Some studies included the 3 A's (ask, advise, arrange quitline referral) [31], counselling using the 5 A's plus NRT [32], brief 'tailored' tobacco advice, assistance using population-specific printed material and NRT [33]. Evidence also showed that behavioural support interventions were effective in improving oral health-related behaviours [34]. Incorporation of tobacco cessation into dental care would provide a unique opportunity for supporting people with diabetes to stop their tobacco use. However, such a facility is very limited in SEA.

Bangladesh is a country in SEA with a high prevalence of diabetes in adults (8.1%). The International Diabetes Federation estimated that 7.1 million people in Bangladesh live with diabetes and almost the same number have undetected diabetes [35]. A recent Global Adult Tobacco Survey (GATS) showed that the prevalence of tobacco use is high in Bangladesh (18% are smokers and 20.6% are ST users) [36]. The level of addiction for ST is high among users [37]. A study estimated that 34% of people with diabetes use tobacco in Bangladesh [38]. Oral diseases are also common among people with diabetes in this country [39]. The increasing prevalence of diabetes and oral diseases, along with high levels of tobacco consumption pose major health challenges in Bangladesh. Furthermore, Bangladesh has no dedicated national tobacco cessation service or quitline [40]. Bangladesh Institute of Research and Rehabilitation for Diabetes, Endocrine and Metabolic Disorders (BIRDEM) is the largest hospital in Bangladesh and is a tertiary hospital with more than 650 beds [41]. People with diabetes from all over the country receive their treatment here. BIRDEM has a dental outpatient department that serves almost 70 patients each day. A tobacco cessation intervention, delivered by dental health professionals in the dental department of BIRDEM, integrated with messages to improve oral hygiene could help people with diabetes quit tobacco and improve their oral health.

Whilst there is currently no evidence of the use of these strategies in a dental setting in Bangladesh with people with diabetes who use tobacco, evidence from other routine health-care settings such as tuberculosis (TB) programmes delivered in primary and secondary care has shown that behavioural support delivered by health workers with one day of training in tobacco cessation, using flipbooks, leaflets and posters was effective in achieving quit rates of 41% at 6 months among people attending TB clinics. This was 4% more effective when combined with pharmacological treatments for cessation [42]. A further study in Bangladesh and Pakistan among TB patients attending healthcare facilities found 25% of people quit at 12 months following brief behavioural advice [43]. It has also been shown that a behavioural change intervention using a flipbook was acceptable and feasible to deliver in the SEA setting for ST cessation [44]. The flipbook consisted of messages based on behaviour change techniques proven to be effective for helping people quit smoking following several stages of behaviour change based on the trans-theoretical model (pre-contemplation, contemplation, preparation, action, and maintenance) [45, 46]. People changing their smoking behaviour focus on different processes at particular stages of change. For example, they use the fewest processes of change during precontemplation; and emphasize raising their consciousness during the contemplation stage. They emphasize self-re-evaluation in both the contemplation and action stages; emphasize self-liberation, a helping relationship, and reinforcement management during the action stage; and use counterconditioning and stimulus control in both action and maintenance stages [47]. In the general population, behavioural support combined with pharmacotherapy is the most effective strategy in helping people to quit smoking [48–50]. No pharmacological therapies, including NRT, are routinely available in Bangladesh, nor has any behavioural change intervention been developed that is tailored to people with diabetes who use tobacco. Therefore, developing such an intervention could be highly beneficial for this population.

When developing a public health intervention, it is vital to engage with the end users and all key stakeholders [51, 52]. Co-production gives the opportunity for the end user to directly shape the intervention and empowers the public to contribute to and draw on research to improve their lives. Co-production increases the involvement of the ultimate beneficiaries and end users of research from the very beginning of the research process. This has the potential to produce stronger research, and research outcomes that better fit the needs, values and interests of end users [53, 54]. Therefore, co-production of a tobacco cessation intervention including adaptation of existing intervention materials to suit a dental setting could be beneficial for this vulnerable group.

The aim of this study was to co-produce a tobacco cessation intervention for people with diabetes for use in dental clinics in Bangladesh. The study had four objectives, as follows:

1. To assess tobacco use (patterns) and perceptions about receiving tobacco cessation support from dentists among people with diabetes attending the dental department of BIRDEM who smoke or use ST.
2. To assess the status of any current tobacco cessation support provision by the dentists at BIRDEM.
3. To assess the barriers and facilitators of implementing a tobacco cessation intervention at BIRDEM.
4. To co-produce a tobacco cessation intervention with people with diabetes, and dentists to be used in the proposed context.

## Methods

**Fig. 1:**
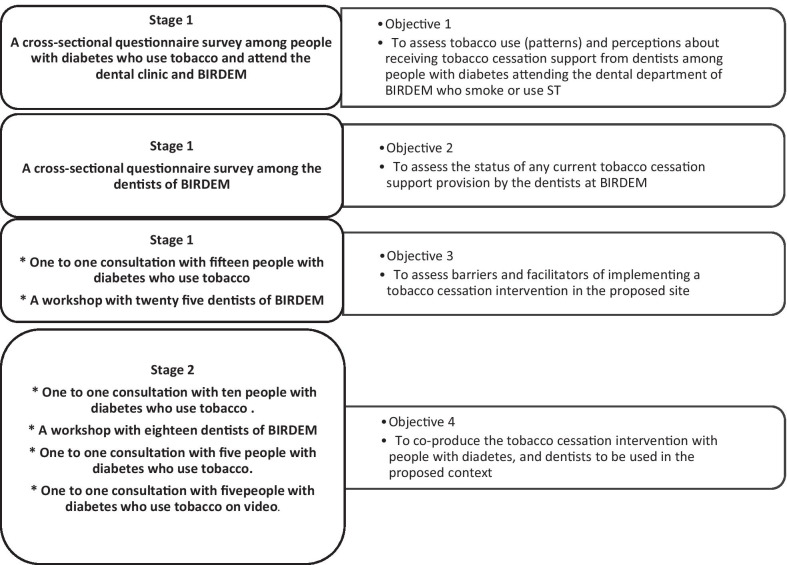
Study objectives and related activity

The study was undertaken in the Department of Dentistry at BIRDEM in Dhaka, Bangladesh. Ethics approval was obtained from the Ethical Review Committee (ERC) of the Diabetic Association of Bangladesh (BADAS). According to phase 1 of the MRC framework for developing and evaluating complex interventions [55], we co-produced the tobacco cessation intervention materials (reported in this paper), with our long-term aim being to test the developed intervention for feasibility, effectiveness and cost-effectiveness (Phases 2–4 of the MRC framework) at a later stage. The study was conducted in two stages adapted from the standard steps of co-production recommended by Hawkins et al. [56]. Stage 1 (related to objective 1, 2 and 3) included surveys, and consultation with people with diabetes attending BIRDEM and workshop with dentists; stage 2 (related to objective 4) involved co-production of the intervention materials (Fig. 1).

Patient and Public Involvement (PPI) activity was embedded at each stage to work collaboratively with the end users and dentists. The research question was developed and informed by the needs and priorities of both key stakeholder groups.

### Stage 1

To assess tobacco use patterns and perceptions of people with diabetes on receiving tobacco cessation support from the dentists, a cross-sectional questionnaire survey was administered between July and August 2019. All people with diabetes (clinically diagnosed as diabetic and registered in BIRDEM) who attended for dental treatment and reported using tobacco were approached to participate in the survey within the two-month data collection period. The survey was conducted among people who agreed to participate. The survey was conducted anonymously, after informed consent was obtained from all participants. The tobacco use questions were adapted from GATS in Bangladesh [36]. Other study specific questions were developed for this study.

To assess the barriers and facilitators of implementing a tobacco cessation intervention, one-to-one consultation was conducted with fifteen people with diabetes who use tobacco. A separate workshop was conducted with twenty-five dentists of BIRDEM to discuss their views about the barriers and facilitators to delivering the tobacco cessation intervention in the dental outpatient department of BIRDEM. Verbal consent was obtained before they participated in the workshop. Notes were taken during the consultation and workshop, and these were summarized in a report. To assess the current tobacco cessation support provision by dentists in this setting, the dentists completed an anonymous questionnaire that was adapted from the National Centre for Smoking Cessation and Training [57] questionnaire for Health Professionals, translated into Bengali. The survey data were analysed using descriptive statistics for the first two objectives. Identification of barriers and facilitators addressed the third objective.

### Stage 2

The second stage of the study was informed by the findings of the first stage and included iterative consultations with patients and a workshop with the dentists (related to the 4th study objective). The aims of these activities were to co-produce the intervention and to outline the training materials for the dentists and dental nurses. The activities of the second stage were conducted in January 2020.

The consultations to co-produce the tobacco cessation intervention included one to one discussion about the intervention components and delivery with ten people with diabetes from BIRDEM who use tobacco. They were asked what support they would like to get from the dental professionals that could help them to stop using tobacco. We planned to develop a brief advice-based tobacco cessation intervention to be delivered by dentists or dental nurses, as suggested by our study participants and supported by information in the literature. To facilitate the consultations, a draft template of a flipbook with different intervention components was shown to them to provoke their ideas and obtain their feedback. We adapted the template from theory-based behaviour change interventions on smoking [43] and smokeless tobacco use among South Asians [44].

A workshop with the dentists working at BIRDEM was undertaken to prioritise messages for the tobacco cessation behaviour support intervention integrated with oral hygiene messages (based on feedback from the participants with diabetes who use tobacco). The workshop also outlined the culturally and contextually suitable intervention components and plans to deliver the intervention in routine dental practice. In this workshop, dentists were asked to respond to the following points for each key message: dentists’ perception of the acceptability to patients; acceptability for the dentists and dental nurses to discuss with patients; perceived effectiveness in helping patients to quit; and feasibility to deliver. They also decided whether each key message should be kept in the behaviour support advice to the patients and training to the dentists and dental nurses or covered in training only but not in the advice session or excluded completely. The participants responded yes/ no for each aspect. The planning of incorporating the intervention within the regular workload, and the potential for incorporation of NRT along with behavioural support were also discussed.

Further refining of the intervention material was conducted with the participants who have diabetes and use tobacco. One to one consultation with another five participants was conducted to show them the intervention materials and obtain their feedback. For the feedback on the delivery plan, a hypothesized study scenario was described, and open questions were used to elicit their response to the delivery plan and invite suggestions. A short video on the harmful effects of tobacco was also created, following suggestions from most of the participants. We received feedback on the short video via consultation with another five participants. The consultation with the patients was analysed focusing on what they thought would work and what would not, and ways to further improve the design. Based on the feedback, we created a tobacco cessation intervention, which combined a behaviour change intervention and pharmacotherapy (NRT). The behaviour change intervention included brief advice using a flipbook, and a short video on the harmful effects of tobacco. We also mapped the major behaviour change techniques (BCTs) with the potential mechanism of action of the key intervention components in the intervention package (based on different stages of behavior change following the ‘transtheoretical model’). BCTs were numbered based on the theory and technique tool [58].

## Results

### Stage 1

A total of 35 people with diabetes who use tobacco and attend the dental outpatient clinic at BIRDEM (20 male and 15 female) completed the survey. Among those eight were cigarette smokers, 20 ST users and 7 dual users. The demographic characteristics of the participants and their tobacco use are presented in Table 1.

**Table 1:** Socio-demographic characteristics of participants with diabetes, their tobacco use and attitude towards tobacco quitting (N, max = 35)

	Categories	n (%)
*Socio-demographic characteristics*		
Sex	Male	20 (57.1)
	Female	15 (42.9)
Age	29–50 years	13 (39.4)
	51–70 years	20 (60.6)
Education	No education	14 (40)
	Primary	8 (22.9)
	Secondary	7 (20)
	Higher	6 (17.1)
Occupation	Service	6 (17.1)
	Household work	14 (40)
	Retired and Unemployed	15 (42.8)
*Tobacco use and attitude towards tobacco quitting*		
Tobacco use	Cigarette	8 (22.9)
	Smokeless tobacco	20 (57.1)
	Dual use	7 (20)
Tobacco use pattern	Daily use	27 (79.4)
	Sometimes	7 (20.6)
Importance of tobacco cessation	No	3 (10)
	Moderately important	11 (35.5)
	Important	7 (22.6)
	Very important	10 (32.3)
Want to quit tobacco	No	6 (17.1)
	Yes, moderate	17 (48.6)
	Yes, a lot	12 (34.3)
Worried about the expense of tobacco	No	23 (65.7)
	Yes, moderate	8 (22.9)
	Yes, a lot	4 (11.4)
Worried about the health effects of tobacco	No	10 (35.7)
	Yes, moderate	12 (42.9)
	Yes, a lot	6 (21.4)
Feel it’s difficult to quit	No	17 (48.6)
	A little	11 (31.4)
	A lot	7 (20)
Tried to quit in last 12 months	Yes	26 (74.3)
	No	9 (25.7)

Their perception about receiving tobacco cessation support from dentists indicated that all participants would like to be asked about their tobacco use and receive support from their dentist about tobacco cessation (Table 2).

**Table 2:** Perception on receiving tobacco cessation support from dentists (N, max = 35)

	Categories	n (%)
If the doctor asked about tobacco use in the past 12 months?	No	20 (57.1)
	Yes	15 (42.9)
If the doctor gave advice for tobacco cessation past 12 months?	No	20 (57.1)
	Yes	15 (42.9)
Would you like it if the dentist asked about your tobacco use?	No	0
	Yes	35 (100)
Would you like it if the dentist gave you short advice on tobacco cessation?	No	0
	Yes	35 (100)
Would you like it if you are requested to set a quit date after giving advice?	No	9 (25.7)
	Yes	26 (74.3)
Would you like it if continuous help/support is provided to you for successful quitting?	No	3 (8.8)
	Yes	31 (91.2)
Would you like if telephone contact is made with you regarding tobacco cessation	No	11 (31.4)
	Yes	24 (68.6)
If you were asked to come again to the dentist in 3 months or 6 months’ time regarding follow-up of tobacco cessation, would you like it?	No	14 (40)
	Yes	21 (60)
Would you like it if you were advised to take a medicine to make tobacco cessation successful?	No	12 (34.3)
	Yes	23 (65.7)

A total of 25 dentists took part in the workshop and 18 (72%) of them considered it was important to provide tobacco cessation advice and support, along with oral hygiene instruction to improve the oral health of people with diabetes. Seven (28%) of them disagreed with that. Currently, 11 (44%) of the dentists always asked their patients about tobacco use and 12 (48%) always advised them about quitting. However, they did not provide any further support. More than 85% did not use any structured or evidence-based approach such as following a flipbook or leaflets or other materials to support their patients to quit tobacco (Table 3).

**Table 3:** The current tobacco cessation support provision by dentists in the site (N, max = 25)

	Categories	n (%)
*How much do the dentists agree with each of the following statements? (1–4)*		
1. I think it is important to provide tobacco cessation advice and support along with oral hygiene instruction to improve the oral health condition of the diabetic population	Disagree	7 (28)
	Moderately agree	5 (20)
	Completely agree	13 (52)
2. With my patients, I always discuss the effects of tobacco on health	Disagree	4 (16)
	Moderately agree	9 (36)
	Completely agree	12 (48)
3. With my patients, I always discuss the effects of tobacco on oral health	Disagree	7 (28)
	Moderately agree	5 (20)
	Completely agree	13 (52)
4. I have enough time and capacity to provide face-to-face tobacco cessation intervention	Disagree	7 (28)
	Moderately agree	14 (56)
	Completely agree	4 (16)
5. I ask my patients about their tobacco use	Always	11 (44)
	Sometimes	13 (52)
	Never	1 (4)
6. I recommend patients to stop using tobacco products	Always	12 (48)
	Sometimes	12 (48)
	Never	1 (4)
7. In my centre, I offer help to patients who want to quit tobacco use	Always	10 (40)
	Sometimes	9 (36)
	Never	6 (24)
8. Do you use any flipbooks or leaflets or other materials to support patients with oral hygiene instruction	Always	1 (4)
	Sometimes	4 (16)
	Never	20 (80)
9. Do you use any flipbooks or leaflets or other materials to support patients to quit tobacco?	Always	0
	Sometimes	3 (14.3)
	Never	18 (85.7)

The initial consultation and workshop reflected that the proposed tobacco cessation intervention delivered within a dental setting in BIRDEM was considered to be important and acceptable by potential end users (people with diabetes who use tobacco and attend the dental department at BIRDEM). The opportunities and barriers identified by both potential end users and dentists are listed in Table 4. In short, willingness of the dentists to help their patients on tobacco cessation and willingness of the patients to receive relevant help from their dentists were identified as the main opportunities. However, not having enough time, and gaps in skills to counsel patients in tobacco cessation, and insufficient resources to support patients in tobacco cessation, were identified as the main barriers.

**Table 4:** Barriers and facilitators identified by dentists and patients for tobacco cessation intervention for diabetic patients at the dental department of BIRDEM

	Barriers	Facilitators
Dentists’ perspective	1. Not having enough time to spend with the patient to give full behaviour change support on tobacco cessation due to work pressure	1. Willingness to help the patients on tobacco cessation
	2. Not having clear knowledge on how to support a patient in tobacco cessation successfully	2. Willingness to learn how they can support their patients on tobacco cessation
	3. Not having a clear understanding of behavioural change theory and interventions	3. Willingness to learn about behavioural change theory and interventions
	4. Not having clear knowledge on nicotine replacement therapy (NRT) and prescribing rules for NRT	4. Willing to learn about NRT for tobacco cessation
	5. No formal structure to take tobacco history from patients	5. Reputed hospital and doctors
	6. No regular/ standard support structure to provide information on harmful effects of tobacco	6. Teachable moment
	7. No regular/ standard support structure for tobacco cessation available	7. As the patients are registered patients with diabetes, it would be possible to track them. Most attend follow-up dental appointments
Patients’ perspective	1. Lack of knowledge of harmful effects of tobacco 2. Misperceptions: believe that ST makes teeth strong 3. Long time addiction; they will find it difficult to quit and relapse when they face negative symptoms while trying to quit 4. Not interested in quitting (especially ST) 5. Have only short time to receive advice 6. Interested in finishing their dental treatment quickly 7. Difficult to come back just to receive advice (if not matched with their other treatment/ follow-up visits)	1. Patients would like their dentist to give advice/help in tobacco cessation 2. Patients are happy to share their phone number 3. Most are willing to come back for follow-up visits if they match with their other scheduled visits

### Stage 2

The co-production activity involved brief advice using a flipbook and video^1^, in combination with pharmacotherapy as this was the intervention that the dentists and potential end users thought would work best in the dental setting. The key intervention components identified be included in the flipbook were: identifying the tobacco products that the patients use; specifying harmful ingredients; emphasizing the adverse health effects of tobacco of tobacco (specifically focusing on the impact of tobacco on oral health, the effect of tobacco on the person’s general health including diabetes); listing the benefits of quitting; dispelling misconceptions/myths; using an ‘importance to quit’ scale; identifying triggers and coping strategies; planning and preparation; establishing readiness to quit; addressing withdrawal symptoms; incorporation of cessation medication along with the behaviour change techniques; and addressing patients’ expectations of cessation medication. In addition, the intervention included oral hygiene instruction. We received positive feedback from the potential end users on the short video (2 min long). They understood the key message of this video about the harmful effects of tobacco on health and found it educational. They found the video motivating to quit tobacco and suggested incorporating that along with the flipbook during the first face-to-face advice. The key intervention components with potential mechanism of action are shown in Table 5 where BCTs were numbered based on the theory and technique tool.

**Table 5:** Major behaviour change techniques (BCT) used and their mechanism of action in the intervention package to support tobacco cessation (based on different stages of behaviour change, based on the ‘transtheoretical model’)

	Pre-contemplation	Contemplation	Preparation	Action	Maintenance
1. BCT used	5.1 Information about health consequences	5.1 Information about health consequences 9.2 Pros and Cons 5.2 Salience of consequences	1.1 Goal setting (behavior) 1.3 Goal Setting (outcome) 1.5 Review behavior goals 1.7 Review outcome goals	1.2 problem solving 12.3 Avoidance/ reducing exposure to cues for the behaviour 11.2 Reduce negative emotions	1.2 problem solving 12.3 Avoidance/ reducing exposure to cues for the behaviour 11.2 Reduce negative emotions
Mechanism of Action	Knowledge	Knowledge, belief about consequences	Goals, beliefs about capability	Beliefs about capability. environmental context and resources, emotion	Beliefs about capability. environmental context and resources, emotion
Content used in flip chart	1.Ingredients used in cigarettes and ST 2. Health effects of tobacco (general health, specific dental health)	1. Ingredients used in cigarettes and ST 2. Health effects of tobacco (general health, specific dental health)	Use of scales: Importance to quit Readiness to quit Confidence to quit Guidance for preparation	1. Guidance for cessation 2. Identify social cues and situations that lead to tobacco use 3.Tips to control the cues 4. Tips to control the withdrawal symptoms	1. Guidance for cessation 2. Identify social cues and situations that lead to tobacco use 3.Tips to control the cues 4. Tips to control the withdrawal symptoms
Content used in video	1.Ingredients used in cigarettes and ST 2. Health effects of tobacco	1.Ingredients used in cigarette and ST 2. Health effects of tobacco			
2. BCT used	3.1 Social support (unspecified)	3.1 Social support (unspecified)	3.2 Social support (practical)	3.1 Social support (unspecified) 3.2 Social support (practical)	3.1 Social support (unspecified) 3.2 Social support (practical)
Mechanism of Action	Environmental context and resources	Environmental context and resources	Social influence	Social influence	Social influence
Content used in flip chart	Financial gain over time not buying tobacco products. Alternative use of saved money	Financial gain over time not buying tobacco products. Alternative use of saved money	Tips to use friends, family and children as sources of encouragement in quitting tobacco	1. Tips to use friends, family and children as sources of encouragement in quitting tobacco 2 Improving public image and boosting confidence to attend social events because of quitting tobacco	1. Tips to use friends, family and children as sources of encouragement in quitting tobacco 2. Improving public image and boosting confidence to attend social events because of quitting tobacco
3. BCT used	3.2 Social support (practical)	3.2 Social support (practical)			
Mechanism of Action	Social influence	Social influence			
Intervention setting	1. Accessing professional support for tobacco cessation 2. Supportive environment at the dental clinic 3. Delivery of the intervention by dentists or dental nurses	1. Accessing professional support for tobacco cessation 2. Supportive environment at the dental clinic 3. Delivery of the intervention by dentists or dental nurses			
Pharmacological				NRT	NRT

## Discussion

The survey results of the people with diabetes attending BIRDEM showed that the majority were smokeless tobacco users, and all were willing to get support for tobacco cessation from their dentist. Whilst a high proportion of dentists reported that they asked and provided advice about tobacco use, the advice was limited to telling their patients to quit, with no further behavioural support or pharmacological interventions to help.

The workshop discussion with the dentists indicated that most did not have access to any resources such as flipbooks, leaflets or other materials, nor did they have appropriate knowledge of techniques to help and support patients to quit using tobacco, but they were willing to provide support. The consultation with potential end users of the intervention and the workshop with the dentists revealed some important facilitators and barriers that need to be considered when developing the intervention. Our study reflected that one of the main barriers of tobacco cessation intervention in the dental setting was lack of time (due to the provision of treatment to a large caseload of patients, dedicating sufficient time to engage in meaningful tobacco cessation consultation becomes infeasible). Lack of resources and training for dentists was identified in our study as another major challenge for providing tobacco cessation support in dental settings. Our study also showed that more than 85% of dentists do not use any structured or evidence-based approach while giving tobacco cessation advice and do not provide any further support. Overall, the intervention seemed to be feasible if it is short and designed to fit in with the activities of the dental department of BIRDEM.

In the second stage of our study, we created a tobacco cessation behaviour support intervention based on brief advice and pharmacotherapy. Following the ‘3 A’ approach: dentists first ask about tobacco use and give very brief advice to the users. They then assist the patient by signposting them to a specially trained dentist or dental nurse who will be designated to provide support using the designed intervention.

Similar to our study findings, lack of both training and resources for dentists were identified as major challenges for providing tobacco cessation support in dental settings in other studies. Prakash et al. (2013) showed that only 39% of dentists assisted with quitting, and 42% dentists had formal training in tobacco cessation in their study of three states in USA [59]. A study in a hospital emergency department in Columbia reported that health care providers missed opportunities for smoking cessation counseling 70% of the time [60]. This situation could be improved by providing proper training to dentists and creating some organizational support structures. Tobacco cessation-related training provision could be incorporated within the standard dental curricula.

Following the evidence of the effectiveness of behavioural support combined with pharmacotherapy in helping people to quit smoking [49] and successful integration of a patient-facing flipbook for brief advice in SEA settings for smoking [43] and ST cessation [44], we considered adopting the same approach. The multi-component tobacco cessation intervention that we created contains (1) a flip book to guide tobacco cessation based on appropriate BCTs, (2) a short video showing the harmful effects of tobacco to increase awareness, and (3) pharmacotherapy (NRT). Other tobacco cessation interventions delivered in dental settings have also used patient-facing flip books, leaflets and videos as intervention components and some studies used NRT [25]. Interventions including advice, assistance, and follow-up support, which are effective for the general population, are expected to be applicable for people with diabetes [20]. In our designed intervention, some health effects were specifically tailored to diabetes and particular oral diseases, as tailoring helps to fully exploit the teachable moment [22]. Exploratory surveys and consultations with participants in our study revealed that they agreed with this approach and considered it acceptable. Therefore, we are expecting to provide the optimum package to help people quit tobacco in the dental setting. We could not include referral to a quit line or support centre within the intervention component as no such services are available in Bangladesh.

The study has some limitations. The survey sample was based on the number of people with diabetes who use tobacco and attended dental department BIRDEM agreed to participate within the two-month data collection period. Secondly, incorporation of a detailed observation study could have identified aspects to inform the health system changes in the current setting and refine the intervention further. Most of the BCTs were adapted from existing studies. However, the BCTs adapted here were validated in South Asian population.

To our knowledge, this is the first study to develop a tobacco cessation intervention for and with people who have diabetes and use tobacco in a dental setting in Bangladesh, using a co-production model. Assessing patients’ perceptions of a tobacco cessation intervention, dentists’ current service provision and identification of barriers and facilitators in this context, will help to find a feasible approach to design a future trial to test the effectiveness of the co-produced intervention materials. Co-production increases the relevance of research by ensuring that it reflects the needs, values and interests of patients and improves the quality of research through broadening the range of expert input. The next stage of work should focus on designing a feasibility trial and training materials. This will create organizational changes to build both a supportive environment for tobacco cessation in the dental setting, and capacity building among dental health professionals.

## Conclusions

As people with diabetes are more prone to oral diseases, and tobacco use has deleterious effects on both conditions, it seems appropriate that the dental professional can make the patient aware of the risks of tobacco use and provide assistance for tobacco cessation. Incorporation of tobacco cessation within dental care provides a unique opportunity to support people with diabetes to stop using tobacco, and hence improving their oral as well as general health. Our co-produced tobacco cessation intervention will be tested for feasibility and effectiveness in a future trial within the dental setting and is expected to be helpful in addressing a major health challenge.

## Data Availability

The datasets used and/or analysed during the current study are available from the corresponding author on reasonable request.

## Footnotes

1 https://www.york.ac.uk/healthsciences/research/public-health/projects/tcdc/tcdc-current-work/.

## References

[1] WHO. WHO global report: mortality attributable to tobacco. WHO. 2012. https://www.who.int/tobacco/publications/surveillance/rep_mortality_attributable/en/. Accessed 3 May 2019.

[2] CDC. Smoking, Gum Disease, and Tooth Loss | Overviews of Diseases/Conditions | Tips From Former Smokers | CDC. https://www.cdc.gov/tobacco/campaign/tips/diseases/periodontal-gum-disease.html. Accessed 22 Aug 2021.

[3] Palmer RM, Wilson RF, Hasan AS, Scott DA (2005). Mechanisms of action of environmental factors—tobacco smoking. J Clin Periodontol.

[4] Leite FRM, Nascimento GG, Scheutz F, López R (2018). Effect of smoking on periodontitis: a systematic review and meta-regression. Am J Prev Med.

[5] Johnson GK, Slach NA (2001). Impact of tobacco use on periodontal status. J Dent Educ.

[6] Reibel J (2003). Tobacco and oral diseases. Update on the evidence, with recommendations. Med Princ Pract.

[7] Stanfill SB, Connolly GN, Zhang L, Jia LT, Henningfield JE, Richter P (2011). Global surveillance of oral tobacco products: total nicotine, unionised nicotine and tobacco-specific N-nitrosamines. Tob Control.

[8] Boffetta P, Hecht S, Gray N, Gupta P, Straif K (2008). Smokeless tobacco and cancer. Lancet Oncol.

[9] Sinha DN, Abdulkader RS, Gupta PC (2016). Smokeless tobacco-associated cancers: a systematic review and meta-analysis of Indian studies. Int J Cancer.

[10] Saeedi P, Petersohn I, Salpea P, Malanda B, Karuranga S, Unwin N (2019). Global and regional diabetes prevalence estimates for 2019 and projections for 2030 and 2045: results from the International Diabetes Federation Diabetes Atlas, 9th edition. Diabetes Res Clin Pract.

[11] Hills AP, Arena R, Khunti K, Yajnik CS, Jayawardena R, Henry CJ (2018). Epidemiology and determinants of type 2 diabetes in south Asia. Lancet Diabetes Endocrinol.

[12] International Diabetes Federation. Diabetes in South-East Asia, IDF Diabetes Atlas. 9th Edition. 2019. https://www.idf.org/our-network/regions-members/south-east-asia/diabetes-in-sea.html. Accessed 24 Nov 2020.

[13] Lamster IB, Lalla E, Borgnakke WS, Taylor GW (2008). The relationship between oral health and diabetes mellitus. J Am Dent Assoc.

[14] González-Serrano J, Serrano J, López-Pintor RM, Paredes VM, Casañas E, Hernández G (2016). Prevalence of oral mucosal disorders in diabetes mellitus patients compared with a control group. J Diabetes Res.

[15] Loe H. Periodontal disease: the sixth complication of diabetes mellitus. In: Diabetes care. American Diabetes Association; 1993. p. 329–34. 10.2337/diacare.16.1.329. 8422804

[16] Mealey BL, Rethman MP (2003). Periodontal disease and diabetes mellitus. Bidirectional relationship. Dent Today.

[17] Taylor GW (2001). Bidirectional interrelationships between diabetes and periodontal diseases: an epidemiologic perspective. Ann Periodontol.

[18] Mirza BAQ, Syed A, Izhar F, Ali Khan A (2010). Bidirectional relationship between diabetes and periodontal disease: review of evidence. J Pak Med Assoc.

[19] Teeuw WJ, Gerdes VEA, Loos BG (2010). Effect of periodontal treatment on glycemic control of diabetic patients: a systematic review and meta-analysis. Diabetes Care.

[20] Haire-Joshu D, Glasgow RE, Tibbs TL (1999). Smoking and diabetes. Diabetes Care.

[21] Register SJ, Harrington KF, Agne AA, Cherrington AL (2016). Effectiveness of non-primary care-based smoking cessation interventions for adults with diabetes: a systematic literature review. Curr Diab Rep.

[22] Lawson PJ, Flocke SA (2009). Teachable moments for health behavior change: a concept analysis. Patient Educ Couns.

[23] Stevens VJ, Severson H, Lichtenstein E, Little SJ, Leben J (1995). Making the most of a teachable moment: a smokeless-tobacco cessation intervention in the dental office. Am J Public Health.

[24] Omaña-Cepeda C, Jané-Salas E, Estrugo-Devesa A, Chimenos-Küstner E, López-López J (2016). Effectiveness of dentist’s intervention in smoking cessation: a review. J Clin Exp Dent.

[25] Holliday R, Hong B, McColl E, Livingstone-Banks J, Preshaw PM (2021). Interventions for tobacco cessation delivered by dental professionals. Cochrane Database Syst Rev.

[26] Carr AB, Ebbert J (2012). Interventions for tobacco cessation in the dental setting. Cochrane Database Syst Rev.

[27] Andrews JA, Severson HH, Lichtenstein E, Gordon JS, Barckley MF (1999). Evaluation of a dental office tobacco cessation program: effects on smokeless tobacco use. Ann Behav Med.

[28] Severson H, Andrews J, Lichtenstein E, Gordon J, Barckley M (1998). Using the hygiene visit to deliver a tobacco cessation program: results of a randomized clinical trial. J Am Dent Assoc.

[29] Severson HH, Peterson AL, Andrews JA, Gordon JS, Cigrang JA, Danaher BG (2009). Smokeless tobacco cessation in military personnel: a randomized controlled trial. Nicotine Tob Res.

[30] Ebbert JO, Carr AB, Patten CA, Morris RA, Schroeder DR (2007). Tobacco use quitline enrollment through dental practices: a pilot study. J Am Dent Assoc.

[31] Gordon JS, Andrews JA, Crews KM, Payne TJ, Severson HH, Lichtenstein E (2010). Do faxed quitline referrals add value to dental office-based tobacco-use cessation interventions?. J Am Dent Assoc.

[32] Binnie VI, McHugh S, Jenkins W, Borland W, MacPherson LM (2007). A randomised controlled trial of a smoking cessation intervention delivered by dental hygienists: a feasibility study. BMC Oral Health.

[33] Gordon JS, Andrews JA, Albert DA, Crews KM, Payne TJ, Severson HH (2010). Tobacco Cessation via public dental clinics: results of a randomized trial. Am J Public Health.

[34] Newton JT, Asimakopoulou K (2015). Managing oral hygiene as a risk factor for periodontal disease: a systematic review of psychological approaches to behaviour change for improved plaque control in periodontal management. J Clin Periodontol.

[35] IDF. International Diabetes Federation. 2020. https://idf.org/our-network/regions-members/south-east-asia/members/93-bangladesh.html. Accessed 18 Sep 2020.

[36] Bangladesh Bureau of Statistics. Global Adult Tobacco Survey Bangladesh. 2017. http://bbs.portal.gov.bd/sites/default/files/files/bbs.portal.gov.bd/page/57def76a_aa3c_46e3_9f80_53732eb94a83/PreliminaryReportonGATSBangladesh2017.pdf. Accessed 14 Oct 2020.

[37] Huque R, Shah S, Mushtaq N, Siddiqi K (2016). Determinants of salivary cotinine among smokeless tobacco users: a Cross-Sectional survey in Bangladesh. PLoS ONE.

[38] Rahman M, Sultana N, Chowdhury Z, CA P. Factors affecting tobacco use among the diabetic patients in Bangladesh. EWMC. 2013;4:1–11. https://www.researchgate.net/profile/Mizanur_Rahman2/publication/256835227_Factors_affecting_Tobacco_Use_among_the_Diabetic_Patients_in_Bangladesh/links/0deec523cfd4ad1a96000000.pdf. Accessed 15 Oct 2020.

[39] Mahbub S, Ferdouse S, Zaman M (2013). Oral health problems of diabetic patients. Bangladesh J Dent Res Educ.

[40] Huque R, Zaman MM, Huq SM, Sinha DN. Smokeless Tobacco and Public Health in Bangladesh. Indian J Public Health. 2017. 10.4103/ijph.IJPH_233_17 PMC6349136 28928314

[41] BIRDEM. BIRDEM General Hospital. 2020. https://www.birdembd.org/. Accessed 15 Oct 2020.

[42] Siddiqi K, Khan A, Ahmad M, Dogar O, Kanaan M, Newell JN (2013). Action to stop smoking in suspected tuberculosis (ASSIST) in Pakistan. Ann Intern Med.

[43] Dogar O, Keding A, Marshall A, Boeckmann DrPH M, Elsey H, Parrott S, et al. Cytisine for smoking cessation in patients with tuberculosis: a multicentre, randomised, double-blind, placebo-controlled phase 3 trial. 2020. www.thelancet.com/lancetgh. Accessed 26 Nov 2020. 10.1016/S2214-109X(20)30312-0 33069301

[44] Siddiqi K, Dogar O, Rashid R, Jackson C, Kellar I, O’Neill N (2016). Behaviour change intervention for smokeless tobacco cessation: its development, feasibility and fidelity testing in Pakistan and in the UK. BMC Public Health.

[45] Prochaska JO (1979). Systems of psychotherapy: a transtheo-retical analysis.

[46] Prochaska JO, Di Clemente CC (1982). Transtheoretical therapy: toward a more integrative model of change. Psychotherapy.

[47] Prochaska JO, DiClemente CC (1983). Stages and processes of self-change of smoking: toward an integrative model of change. J Consult Clin Psychol.

[48] Roberts NJ, Kerr SM, Smith SMS (2013). Behavioral interventions associated with smoking cessation in the treatment of tobacco use. Heal Serv Insights.

[49] Stead LF, Perera R, Bullen C, Mant D, Hartmann-Boyce J, Cahill K (2012). Nicotine replacement therapy for smoking cessation. Cochrane Database Syst Rev.

[50] Hartmann-Boyce J, Chepkin SC, Ye W, Bullen C, Lancaster T. Nicotine replacement therapy versus control for smoking cessation. Cochrane Database Syst Rev. 2018;2018. 10.1002/14651858.CD000146.pub5 PMC6353172 29852054

[51] Roberts L, Turner K, George’ S, Ward D. Briefing notes for researchers: public involvement in NHS, public health and social care research. 2012. http://www.invo.org.uk/wp-content/uploads/2012/04/INVOLVEBriefingNotesApr2012.pdf. Accessed 9 Oct 2020.

[52] Staniszewska S, Denegri S (2013). Patient and public involvement in research: future challenges. Evid Based Nurs.

[53] Forsythe LP, Carman KL, Szydlowski V, Fayish L, Davidson L, Hickam DH (2019). Patient engagement in research: early findings from the patient-centered outcomes research institute. Health Aff.

[54] Brett J, Staniszewska S, Mockford C, Herron-Marx S, Hughes J, Tysall C (2014). Mapping the impact of patient and public involvement on health and social care research: a systematic review. Heal Expect.

[55] Craig P, Dieppe P, Macintyre S, Michie S, Nazareth I, Petticrew M (2008). Developing and evaluating complex interventions: the new Medical Research Council guidance. BMJ.

[56] Hawkins J, Madden K, Fletcher A, Midgley L, Grant A, Cox G (2017). Development of a framework for the co-production and prototyping of public health interventions. BMC Public Health.

[57] NCSCT. National Centre for Smoking Cessation and Training. 2020. https://www.ncsct.co.uk/. Accessed 25 Dec 2020.

[58] The Theory and Techniques Tool. The Theory and Techniques Tool. The Human Behaviour Change Project. 2020. https://theoryandtechniquetool.humanbehaviourchange.org/tool. Accessed 11 Dec 2020.

[59] Prakash P, Belek MG, Grimes B, Silverstein S, Meckstroth R, Heckman B (2013). Dentists’ attitudes, behaviors, and barriers related to tobacco-use cessation in the dental setting. J Public Health Dent.

[60] Buchbinder M, Wilbur R, Zuskov D, McLean S, Sleath B (2014). Teachable moments and missed opportunities for smoking cessation counseling in a hospital emergency department: a mixed-methods study of patient-provider communication. BMC Health Serv Res.

